# Mortality and complications after percutaneous endoscopic gastrostomy: a retrospective multicentre study

**DOI:** 10.1186/s12876-022-02429-0

**Published:** 2022-07-28

**Authors:** K. Stenberg, A. Eriksson, C. Odensten, D. Darehed

**Affiliations:** 1 Department of Surgery, Sunderby Hospital, Kirurgkliniken, Sunderby sjukhus, Sjukhusvägen 10, 954 42 Södra Sunderbyn, Sweden; 2 Department of Surgical and Preoperative Sciences, Surgery, Sunderby Research Unit, Umeå University, Umeå, Sweden; 3 Department of Public Health and Clinical Medicine, Sunderby Research Unit, Umeå University, Umeå, Sweden

**Keywords:** Percutaneous endoscopic gastrostomy, Postoperative complications, Endoscopic surgery, Therapeutic endoscopy, Mortality, Outcomes

## Abstract

**Background:**

Percutaneous endoscopic gastrostomy (PEG) is the method of choice for patients in need of long-term nutritional support or gastric decompression. Although it is considered safe, complications and relatively high mortality rates have been reported. We aimed to identify risk factors for complications and mortality after PEG in routine healthcare.

**Methods:**

This retrospective study included all adult patients who received a PEG between 2013 and 2019 in Region Norrbotten, Sweden.

**Results:**

389 patients were included. The median age was 72 years, 176 (45%) were women and 281 (72%) patients received their PEG due to neurological disease. All-cause mortality was 15% at 30 days and 28% at 90 days. Malignancy as the indication for PEG was associated with increased mortality at 90 days (OR 4.41, 95% CI 2.20–8.88). Other factors significantly associated with increased mortality were older age, female sex, diabetes mellitus, heart failure, lower body mass index and higher C-reactive protein levels. Minor and major complications within 30 days occurred in 11% and 15% of the patients, respectively. Diabetes increased the risk of minor complications (OR 2.61, 95% CI 1.04–6.55), while those aged 75 + years were at an increased risk of major complications, compared to those younger than 65 years (OR 2.23, 95% CI 1.02–4.85).

**Conclusions:**

The increased risk of death among women and patients with malignancy indicate that these patients could benefit from earlier referral for PEG. Additionally, we found that age, diabetes, heart failure, C-reactive protein and body mass index all impact the risk of adverse outcomes.

## Background

Percutaneous endoscopic gastrostomy (PEG) was first introduced in 1980 and has since become the method of choice for patients in need of long-term nutritional support or gastric decompression [1, 2]. The main indications for PEG placement are need for enteral nutrition in patients with neurological disease, obstructive cancer or trauma causing inadequate oral intake due to dysphagia or obstruction, or need for stomach decompression in patients with malignant bowel obstruction [2]. PEG for nutritional support is not commonly recommended if the patient’s life expectancy is less than 30 days [3–5]. Some of the advantages of PEG compared to open surgery for gastrostomy is that it does not require general anaesthesia and that previous studies have shown lower complication rates [6, 7]. A recent meta-analysis found that patients who received PEG have lower complication rates and most likely a higher quality of life than patients with a nasogastric tube, although there was no significant difference in mortality rates [8].

Since PEG was first introduced, two different methods have evolved: the introducer method and the pull method (also known as Gauderer-Ponsky) [2]. With the introducer method, the gastrostomy tube is pushed through the abdominal wall into the ventricle using an introducer kit while the ventricle is endoscopically inflated with gas. With the pull method, the gastrostomy tube is pulled past the patients’ pharynx and oesophagus and then from the inside of the ventricle out through the abdominal wall over a wire. Later studies have shown a lower complication rate in the pull method; therefore, it is the method of choice **i**when technically possible [9–12].

Although PEG is considered a relatively safe and effective method for inserting a gastric tube, previous studies have shown that minor complications (for example local infection, local bleeding, leakage around the stoma, dislocation of the tube, tube blockage and local pain) occur in 18%–38% of all patients, major complications (for example, aspirational pneumonia, organ damage, major bleeding and peritonitis) occur in 2%–4% and the 30-day mortality is reported to be between 3 and 23% [13–19]. The risk of complications after PEG has been found to be increased in patients with other medical conditions such as diabetes mellitus and malignancy and among patients with anti-coagulant therapy or patients treated with corticosteroids [18, 20, 21].

Apart from the risk of complications, some studies have found associations between 30-day mortality and high serum C-reactive protein (CRP) levels, low serum albumin levels, low serum thrombocyte levels, anaemia, old age, chronic obstructive lung disease, ischemic heart disease, underlying neurological disease and recent treatment in an intensive care unit, although the findings are not consistent [13–23]. Due to the conflicting results of previous studies we aimed to study comorbidities and outcomes in patients undergoing a PEG procedure in a routine clinical setting. Our main hypothesis was that the indication for the procedure could impact both mortality and complication rates, with secondary hypotheses that the procedure method, the presence of comorbidities and basic laboratory tests all contribute to the risk for adverse outcomes; hence, our goal was to investigate which factors had an impact on the outcomes, and how large the effect was.

## Methods

### Settings

We conducted this study in Region Norrbotten, a region in the northernmost part of Sweden with approximately 250 000 inhabitants. Primary care is provided through 28 primary care centres spread across the region, while secondary care is provided at five hospitals located in Kiruna, Gällivare, Sunderbyn, Kalix and Piteå. PEG is only conducted in two of these hospitals, Gällivare and Sunderby hospital. The procedure is performed by approximately 30 different surgeons and surgical residents in Region Norrbotten. The procedure is mostly performed in an endoscopic ward in sedation not requiring an anaesthesiologist present, however with the possibility of doing it in general anaesthesia in an operation theatre when needed. Patients routinely receive a single dose of prophylactic antibiotics given through the gastrostomy and the end of the procedure. Standard diameter of the PEG tube used in Region Norrbotten is 20 French.

### Study design and data collection

In this retrospective study we used a regional administrative database (Datalagret) to find patients in Region Norrbotten who had undergone a PEG procedure between 2013 and 2019. To this end, we searched the database for all patients diagnosed with the ICD-10 code JDB10 (percutaneous endoscopic gastrostomy). Patients younger than 18 years were excluded. Patient characteristics, all-cause mortality within 30 and 90 days, cause of death and if any complications had occurred within 30 days of the procedure were then collected from their medical records. In Region Norrbotten all hospitals and almost all primary health care providers use the same medical record system which enabled a broad search for background data including laboratory tests and comorbidities. To facilitate comparison with previous studies, complications were subdivided into minor and major complications**.**

### Variables and statistical methods

Our primary outcome was all-cause mortality at 30 days, with secondary outcomes including mortality at 90 days, as well as minor- and major complications occurring within 30 days from the procedure. Minor complications included accidental dislocation of gastrostomy tube, local infection, leakage around stoma, abdominal pain requiring surgical consultation or tube blockage. Major complications included aspirational pneumonia, organ damage, major bleeding requiring blood transfusion, intraabdominal leakage requiring surgery or peritonitis requiring surgical consultation. All outcomes were measured as dichotomous variables (yes/no). Our main exposure variable was the indication for the PEG procedure, classified into three categories: neurological (such as stroke, Parkinson disease, motor neuron disease etcetera), malignancy (any tumour obstruction proximal to the ventricle causing need for enteral nutrition or tumour obstruction distal to the ventricle causing need for gastric decompression) and other causes of dysphagia (trauma, infections, unknown dysphagia etcetera). Other variables that were screened for having an impact on the outcomes included age, sex, body mass index (BMI), method (introducer method vs pull method), which hospital performed the procedure, comorbidity (diabetes mellitus, heart failure, ischemic heart disease, chronic obstructive pulmonary disease, anticoagulation therapy) and laboratory tests (haemoglobin (Hb), CRP, thrombocytes, albumin level and creatinine level) measured closest prior to the procedure but up to a maximum of 30 days in advance.

We analysed patient characteristics and missing data, followed by unadjusted and adjusted analyses. Patient characteristics and missing data are presented as absolute numbers and proportions. In the unadjusted analyses all scale variables were transformed to the ordinal scale for better visualization. Missing data were handled by using multiple imputation, using all confounders and outcomes as predictors while only imputing confounders. Two variables that was planned for inclusion were excluded; the first being albumin, which was excluded from all further analyses due to 58% missing data, the second being treatment with a higher than prophylactic dose of low-molecular-weight heparin (anticoagulation therapy), where only four patients (1%) in the study group were treated with a higher dose and hence it was also excluded from further analyses. Finally, it should be noted that there were 378 unique patients, but 11 of these received a PEG twice during the study period meaning that 389 PEG operations were conducted. We concluded that each PEG operation was valuable to include, and since the number of patients receiving a PEG twice was low, we chose to use the term “patients” throughout the manuscript.

We used multivariable logistic regression analyses to adjust for confounding factors. Multicollinearity was addressed by constructing a matrix for bivariate correlations as well as addressing variance inflation factor statistics. The assumption of linearity was tested using the Box-Tidwell test. Age, thrombocytes, and creatinine did not fulfil the assumption for all outcomes and hence was transformed to the ordinal scale in the final model for this outcome; otherwise, the models were built identically for all outcomes. The following variables were included in the final multivariable models: indication, year of procedure, hospital, operation method, age, sex, BMI, diabetes, heart failure, ischemic heart disease, chronic obstructive pulmonary disease, CRP, Hb, thrombocytes and creatinine levels.

All confounders were chosen based on clinical experience and probability of availability in the medical records. The robustness of the multivariable analyses was checked by performing analyses only on patients undergoing their first PEG operation which showed similar results, and as complete-case analyses on unimputed data which showed similar but attenuated results. All variable and the modelling strategy were decided a priori. We performed all statistical analyses in IBM SPSS Statistics 26. Ethical approval was obtained from the Swedish Ethical Review Authority (DNR 2020-01,917).

## Results

### Patient characteristics and missing data

We found a total of 393 patients. Four patients were excluded because of age under 18 years, which left a total of 389 patients, of whom all were included in the study. In all, 176 (45%) were women, the median age was 72 years, and the median BMI was 22. Ischemic heart disease was the most common comorbidity, occurring in 80 (21%) patients. In all, 261 (73%) patients received their gastrostomy using the pull method, and the most common indication for the procedure was neurological disease, which affected 281 (72%) patients. Most of the variables had no missing data, and among variables with missing data, the level was generally low. Baseline data and patient characteristics are shown in Table 1.

**Table 1:** Baseline data and patient characteristics

	Total sample, n (%)	Missing, N (%)
All patients	389	
Indication Neurologic disease Malignancy Other dysphagia	281 (72%) 80 (21%) 28 (7%)	0
Operation method Pull Introducer	261 (67%) 95 (24%)	33 (8%)
Age, median (q1–q3)	72 (63–79)	0
Female sex	176 (45%)	0
Body mass index, median (q1–q3)	22 (20–25)	38 (10%)
Diabetes	63 (16%)	0
Heart failure	47 (12%)	0
Ischemic heart disease	80 (21%)	0
Chronic obstructive pulmonary disease	33 (8%)	0
Anticoagulation treatment	4 (1%)	0
C-reactive protein, mg/l, median (q1-q3)	24 (9–55)	60 (15%)
Haemoglobin, g/l, median (q1-q3)	123 (110–133)	40 (10%)
Thrombocytes, 10^9^/l median (q1-q3)	290 (220–376)	40 (10%)
Albumin, g/l, median (q1-q3)	31 (27–36)	226 (58%)
Creatinine, µmol/l, median (q1-q3)	56 (45–70)	47 (12%)

### Mortality

**Fig. 1:**
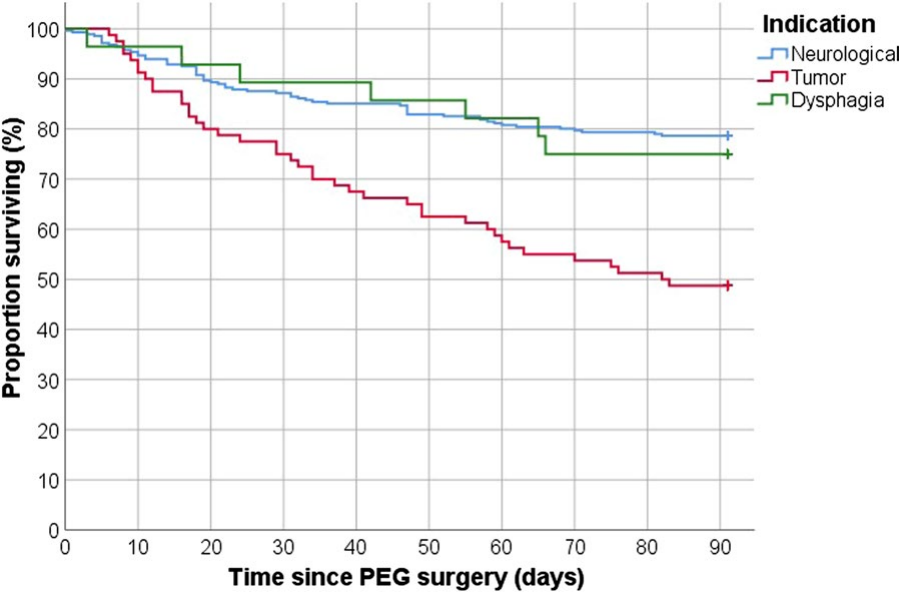
Kaplan–Meier Curve of the proportion surviving within 90 days depending on the indication for the PEG procedure

Mortality was 15% (n = 59) at 30 days and 28% (n = 108) at 90 days. The most common cause of death was malignancy, followed by aspiration pneumonia (Table 2). The unadjusted analyses revealed a much higher mortality rate at both 30 and 90 days for patients where the indication for PEG was malignancy (25% at 30 days and 51% at 90 days) compared to neurological deficit (13% at 30 days and 21% at 90 days) (Table 3). A visualization of this is provided as a Kaplan–Meier curve (Fig. 1). In the adjusted analyses malignancy as indication was significantly associated with higher mortality at 90 days (Odds Ratio [OR] 4.41, 95% Confidence interval [CI] 2.20–8.88) (Table 4). Every year increase in age significantly increased mortality at 30 days (OR 1.03 95% CI 1.01–1.06). Each point increase in CRP significantly increased the mortality rate at 30 days (OR 1.01, 95% CI 1.00–1.01) and at 90 days (OR 1.01, 95% CI 1.00–1.01). Mortality at 90 days was significantly higher for women (OR 1.95, 95% CI 1.11–3.44) while it significantly decreased with increasing BMI (OR 0.91 per point, 95% CI 0.85–0.98). Mortality at 90 days was also significantly higher if the patient had diabetes (OR 2.11, 95% CI 1.07–4.19) or heart failure (OR 2.29 95% CI 1.02–5.14). There was also a significant difference in mortality between the two hospitals at both 30 days (OR 0.18 95% CI 0.08–0.41) and 90 days (OR 0.39 CI 95% 0.19–0.78). Tables 3 and 4 shows a full account of the unadjusted and adjusted analyses.

**Table 2:** All-cause mortality and causes of death

	< 30 days, N (%)	< 90 days, N (%)
All-cause mortality, N (% of total sample)	59 (15%)	108 (28%)
Cause of death, N (% of total mortality)		
Malignancy	17 (29%)	37 (34%)
Aspiration pneumonia	15 (25%)	24 (22%)
Motor neuron disease	7 (12%)	11 (10%)
Pneumonia including COPD* exacerbation	5 (8%)	11 (10%)
Stroke	5 (8%)	5 (5%)
Myocardial infarction	1 (2%)	2 (2%)
Other causes**	4 (7%)	7 (6%)
Unknown cause	5 (8%)	11 (10%)

*Chronic obstructive pulmonary disease

**Other causes include major bleeding, extremity ischemia, peritonitis, sepsis, and pulmonary embolism

**Table 3:** Unadjusted analyses

	Death < 30 days, N (%)	Death < 90 days, N (%)	Minor complication, N (%)	Major complication, N (%)	Total sample, N
Indication					
Neurological	36 (13%)	60 (21%)	25 (9%)	47 (17%)	281
Malignancy	20 (25%)	41 (51%)	10 (13%)	7 (9%)	80
Other dysphagia	3 (11%)	7 (25%)	5 (18%)	2 (7%)	28
Operation method					
Pull	36 (14%)	70 (27%)	22 (8%)	37 (14%)	261
Introducer	16 (17%)	27 (28%)	15 (16%)	13 (14%)	95
Age					
18–64	10 (9%)	21 (18%)	11 (10%)	14 (12%)	115
65–74	14 (13%)	28 (25%)	17 (15%)	10 (9%)	111
75 +	35 (22%)	59 (36%)	12 (7%)	32 (20%)	163
Sex					
Women	28 (16%)	53 (30%)	20 (11%)	25 (14%)	176
Men	31 (15%)	55 (26%)	20 (9%)	31 (15%)	213
Diabetes mellitus					
No	46 (14%)	83 (26%)	31 (10%)	43 (13%)	326
Yes	13 (21%)	25 (40%)	9 (14%)	13 (21%)	63
Heart failure					
No	47 (14%)	88 (26%)	39 (11%)	48 (14%)	342
Yes	12 (26%)	20 (43%)	1 (2%)	8 (17%)	47
Ischemic heart disease					
No	41 (13%)	77 (25%)	32 (10%)	43 (14%)	309
Yes	18 (23%)	31 (39%)	8 (10%)	13 (16%)	80
COPD*					
No	55 (15%)	98 (28%)	37 (10%)	50 (14%)	356
Yes	4 (12%)	10 (30%)	3 (9%)	6 (18%)	33
Body mass index (KG/M^2^)					
< 18.5	10 (15%)	22 (33%)	9 (13%)	12 (18%)	67
18.5–24.9	32 (16%)	60 (30%)	21 (10%)	27 (13%)	202
25.0–29.9	9 (15%)	13 (22%)	6 (10%)	10 (17%)	59
30 +	1 (4%)	3 (13%)	0 (0%)	4 (17%)	23
C-reactive protein (MG/L)					
< 10	8 (9%)	13 (15%)	8 (9%)	7 (8%)	86
10–49	24 (16%)	44 (30%)	14 (10%)	24 (16%)	147
50–99	15 (23%)	28 (43%)	4 (6%)	16 (25%)	65
100 +	10 (32%)	16 (52%)	2 (7%)	6 (19%)	31
Haemoglobin (G/L)					
< 100	8 (24%)	17 (50%)	2 (6%)	7 (21%)	34
100–119	24 (22%)	37 (34%)	11 (10%)	21 (19%)	109
120–134	12 (10%)	28 (23%)	12 (10%)	14 (11%)	124
135 +	15 (18%)	23 (28%)	7 (9%)	13 (16%)	82
Thrombocytes (X10^9^/L)					
< 150	4 (22%)	9 (50%)	2 (11%)	0 (0%)	18
150–349	37 (16%)	62 (27%)	20 (9%)	39 (17%)	231
350 +	18 (18%)	34 (34%)	10 (10%)	16 (16%)	100
Creatinine (ΜMOL/L)					
< 50	18 (13%)	39 (29%)	9 (7%)	20 (15%)	134
50–89	31 (17%)	52 (29%)	19 (11%)	30 (17%)	178
90 +	10 (33%)	13 (43%)	3 (10%)	5 (17%)	30

*Chronic obstructive pulmonary disease

**Table 4:** Adjusted analyses

	Odds ratio (95% CI*)
Mortality at 30 days	
Age (per year)	1.03 (1.01–1.06)
C-reactive protein (per point)	1.01 (1.00–1.01)
Hospital 2	0.18 (0.08–0.41)
Mortality at 90 days	
Indication = malignancy**	4.41 (2.20–8.88)
Female sex	1.95 (1.11–3.44)
Diabetes	2.11 (1.07–4.19)
Heart failure	2.29 (1.02–5.14)
Body mass index (per point)	0.91 (0.85–0.98)
C-reactive protein (per point)	1.01 (1.00–1.01)
Hospital 2	0.39 (0.19–0.78)
Minor complication	
Diabetes	2.61 (1.04–6.55)
Major complication	
Age 75 years or older***	2.23 (1.02–4.85)
Hospital 2	0.42 (0.19–0.91)

Only significant results are shown

*Confidence interval

**Reference indication is neurological disease

***Reference age is 18–64 years

### Complications

Minor complications were seen in 41 (11%) patients, and major complications were seen in 57 (15%) patients. The most common minor complications were dislocation of the tube (49%) and local infection (30%), while the most common major complications were aspiration pneumonia (74%) and organ damage (9%). If aspiration pneumonias are excluded, the total number of major complications is 15 (4%) (Table 5). In the adjusted analyses diabetes significantly increased the risk for minor complications (OR 2.61, 95% CI 1.04–6.55), while there was a significantly higher risk for major complications among patients aged 75 years or older, compared to those younger than 65 years (OR 2.23, 95% CI 1.02–4.85). Finally, we also found a significant difference in the risk of major complications between the two hospitals (OR 0.42, 95% CI 0.19–0.91) (Table 4).

**Table 5:** List of complications

	N (%)
Minor complications:	
Dislocation of tube	20 (49%)
Local infection	12 (29%)
Leakage around stoma	5 (12%)
Abdominal pain	2 (5%)
Minor bleeding	1 (2%)
Tube blockage	1 (2%)
Total*, N (% of total sample)	41 (11%)
Major complications:	
Aspiration pneumonia	42 (74%)
Organ damage	5 (9%)
Major bleeding requiring transfusion	4 (7%)
Intraabdominal leakage requiring surgery	3 (5%)
Peritonitis	3 (5%)
Total*, N (% of total sample)	57 (15%)

*One patient had two minor complications, one patient had one minor and one major complication, and one patient had two major complications. Hence the total number of patients suffering complications after the PEG were 95

## Discussion

We found that malignancy as indication for PEG was significantly associated with higher mortality at 90 days after the procedure, compared to neurological disease as indication. We also found several other factors that were associated with higher mortality rates and more complications, including older age, female sex, diabetes, heart failure, increasing CRP levels and lower BMI.

The finding in our study that patients with malignancy as indication for PEG is significantly associated with increased mortality is in line with several previous studies [18, 24, 25] although in this study we did not differ between malignant bowel obstruction and malignancy causing inadequate oral nutrition. Compared to previous studies, the proportion of patients with malignancy in our study was much lower than that of patients with neurological disease. This is probably explained by differences in the criteria for referral in different regions of Sweden as well as in other countries, i.e., patients with malignancy are probably referred for PEG procedures more rarely and later in Region Norrbotten, compared to patients in previous studies. There was no significant difference in the complication rates between the two groups, indicating that the higher mortality rate is a consequence of greater comorbidity and frailty among patients with malignancy, rather than a consequence of the procedure itself. Earlier referral for patients with malignancy in need for PEG would probably benefit these patients, as PEG has in previous studies been showed to increase quality of life compared to a nasogastric tube [8].

Furthermore, low BMI as a risk factor for mortality at 90 days also indicate that many of our patients would benefit from PEG at an earlier stage of their disease, since the indication for PEG in many cases is to achieve sufficient nourishment. Our finding that higher CRP levels are associated with worse outcomes is not surprising, has been shown in previous studies, and should be considered an indication of a more severe underlying condition [15, 17, 22, 23]. The finding that underlying diabetes and heart failure increases the risk of mortality has also been confirmed in previous studies [20, 21, 23].

The higher mortality rate among women was not something that we expected to find and has not been reported in previous studies. In all, 55% of the patients in our study were men, which is in line with previous studies, showing that men account for 56%–71% of all included patients [13, 14, 18, 20, 23]. This indicates that men are referred and accepted for PEG more often. Probably, there are more women who could benefit from PEG but are either never referred for the procedure or are denied more often than men are. This is something that warrants further research.

While the frequency of minor complications was somewhat lower compared with previous studies, there was a surprisingly high number of major complications in our study. An explanation for this is the high rate of aspirational pneumonias. However, aspiration pneumonias might be a complication of the underlying condition which indicated the procedure, as well as a complication of the procedure itself, and hence it could be discussed whether it should be measured as a complication or as a result of the underlying disease. In this study, we included all reported aspirational pneumonias as a major complication, and hence the high number of major complications should be interpreted with care, although clinicians should be aware of the risk of aspiration pneumonias regardless of the cause of it. If we excluded aspiration pneumonias as a complication, the number of major complications would be 15 (4%), which is more in line with previous studies.

There was a large difference in outcomes between the two hospitals in our study, where one hospital performed better both regarding mortality and major complications. Given that the equipment, routines and to some extent the surgeons do not vary between the two hospitals, we believe that the difference is probably explained by differences in patient referral patterns. This is supported by the fact that the hospital with a significantly higher mortality rate performed much fewer PEG procedures than expected given the population in the catchment area. These findings together with the findings of the higher mortality rates for patients with malignancy and among women have been reported back to the hospitals which are now improving local referral routines with the goal of a more equal care between the hospitals.

A major strength of this study is the wide inclusion criteria where we included all adult patients in an entire region in Sweden, which limits inclusion bias. However, being a retrospective study, it has some obvious flaws such as the absence of a standard protocol for patient follow-up. We believe this limits our understanding of complications, especially for minor complications, a theory supported by the fact that we found fewer of these than expected when comparing our results to previous studies. Death, however, is very robustly reported in medical records, minimizing bias regarding this. Another flaw is our understanding of the preoperative status among the patients, preferably we would have had information on for example ASA (American Society of Anesthesiologists) class, as well as a more standardised measurement of comorbidities using for example the Charlson Comorbidity Index, as presented in a previous study on PEG [26]. This data was however not collected since it would have required searches in other databases which we had no access to. The external validity of our study is questionable due to the possible differences in referral patterns in our study compared with previous studies as well as other regions. However, we believe our study make a good contribution to the understanding of complications and mortality after PEG procedures in a routine clinical setting, especially since our study includes two different hospitals and a relatively large number of patients.

## Conclusion

The increased risk of death among women and patients with malignancy indicate that these patients could benefit from earlier referral for PEG. We also found that older age, diabetes, heart failure, C-reactive protein level and body mass index impact the risk of adverse outcomes, all of which could be considered in preoperative PEG risk assessment in routine healthcare. Finally, we found significant differences in outcomes between the two hospitals, and an initiative to equalize care has started. We believe similar differences could be present in other regions and countries, and by highlighting this we hope to inspire others to investigate their own setting with the goal of improving care for patients in need of PEG.

## Data Availability

The datasets generated and analysed during the current study are not publicly available due Swedish Regulations but are available from the corresponding author on reasonable request and they will then be anonymised before sharing.

## Abbreviations

PEG: Percutaneous endoscopic gastrostomy
CI: Confidence interval
OD: Odds ratio
CRP: C-reactive protein
BMI: Body mass index
Hb: Haemoglobin
